# Prioritising Cochrane reviews to be updated with health equity focus

**DOI:** 10.1186/s12939-023-01864-z

**Published:** 2023-05-05

**Authors:** Eve Tomlinson, Jordi Pardo Pardo, Torunn Sivesind, Mindy D Szeto, Melissa Laughter, Ruth Foxlee, Michael Brown, Nicole Skoetz, Robert P Dellavalle, Juan VA Franco, Mike Clarke, Alison Krentel, Ludovic Reveiz, Ashrita Saran, Frances Tse, George A Wells, Robert Boyle, Jennifer Hilgart, Euphrasia Ebai-Atuh Ndi, Vivian Welch, Jennifer Petkovic, Peter Tugwell

**Affiliations:** 1 Population Health Sciences, Bristol Medical School, University of Bristol, Bristol, UK; 2 Faculty of Medicine, School of Epidemiology and Public Health, University of Ottawa, Ottawa, Canada; 3 Ottawa Methods Centre, Ottawa Hospital Research Institute, Ottawa, Canada; 4 Department of Dermatology, University of Colorado School of Medicine, Aurora, CO USA; 5 Department of Dermatology, University of Colorado Anschutz Medical Campus, Aurora, CO USA; 6 The Ronald O. Perelman Department of Dermatology, New York University School of Medicine, New York, USA; 7 Cochrane, London, UK; 8 Michigan State University College of Human Medicine, Grand Rapids, MI USA; 9 Evidence-based Medicine, Department I of Internal Medicine, Faculty of Medicine, University Hospital Cologne, University of Cologne, Cologne, Germany; 10 University of Colorado School of Medicine, Aurora, CO USA; 11 Dermatology Service, Department of Veterans Affairs, Eastern Colorado Health Care System, Aurora, CO USA; 12 Institute of General Practice, Medical Faculty of the Heinrich-Heine-University Düsseldorf, Düsseldorf, Germany; 13 Cochrane Methodology Review Group; Queen’s University Belfast, Royal Hospitals, Grosvenor Road, BT12 6BJ Belfast, UK; 14 School of Epidemiology and Public Health, University of Ottawa, Ottawa, Canada; 15 Bruyere Research Institute, Ottawa, Canada; 16 Knowledge Translation Program, Evidence and Intelligence for Action in Health Department, Pan American Health Organization, Washington, DC USA; 17 Campbell South Asia, New Delhi, India; 18 Division of Gastroenterology, Department of Medicine, McMaster University, Hamilton, ON Canada; 19 National Heart and Lung Institute, Imperial College London, London, UK; 20 Evidence Production & Methods Directorate, Cochrane Central Executive Team, London, UK; 21 Cameroon Consumer Service Organisation (CamCoSO), Bamenda, Cameroon

**Keywords:** Systematic review, Health equity, Priority setting, Prioritisation, Cochrane

## Abstract

**Background:**

The prioritisation of updating published systematic reviews of interventions is vital to prevent research waste and ensure relevance to stakeholders. The consideration of health equity in reviews is also important to ensure interventions will not exacerbate the existing inequities of the disadvantaged if universally implemented. This study aimed to pilot a priority setting exercise based on systematic reviews of interventions published in the Cochrane Library, to identify and prioritise reviews to be updated with a focus on health equity.

**Methods:**

We conducted a priority setting exercise with a group of 13 international stakeholders. We identified Cochrane reviews of interventions that showed a reduction in mortality, had at least one Summary of Findings table and that focused on one of 42 conditions with a high global burden of disease from the 2019 WHO Global Burden of Disease report. This included 21 conditions used as indicators of success of the United Nations Universal Health Coverage in attaining the Sustainable Development Goals. Stakeholders prioritised reviews that were relevant to disadvantaged populations, or to characteristics of potential disadvantage within the general population.

**Results:**

After searching for Cochrane reviews of interventions within 42 conditions, we identified 359 reviews that assessed mortality and included at least one Summary of Findings table. These pertained to 29 of the 42 conditions; 13 priority conditions had no reviews with the outcome mortality. Reducing the list to only reviews showing a clinically important reduction in mortality left 33 reviews. Stakeholders ranked these reviews in order of priority to be updated with a focus on health equity.

**Conclusions:**

This project developed and implemented a methodology to set priorities for updating systematic reviews spanning multiple health topics with a health equity focus. It prioritised reviews that reduce overall mortality, are relevant to disadvantaged populations, and focus on conditions with a high global burden of disease. This approach to the prioritisation of systematic reviews of interventions that reduce mortality provides a template that can be extended to reducing morbidity, and the combination of mortality and morbidity as represented in Disability-Adjusted Life Years and Quality-Adjusted Life Years.

**Supplementary Information:**

The online version contains supplementary material available at 10.1186/s12939-023-01864-z.

## Introduction

Systematic reviews provide a useful source of synthesised evidence to inform health decision making. They are influential to support health technology assessment, guideline development, selection of essential medicines and policy making [1]. Cochrane is an international organisation producing high quality, relevant and up-to-date systematic reviews. Cochrane was initially primarily ‘supply-driven’ with clinician leaders undertaking rigorous methodology pioneered by Iain Chalmers and colleagues in foundational Pregnancy and Childbirth reviews. Since then, Cochrane Review Groups (CRGs) have increasingly moved to being ‘needs-driven’ with targeted research programs based on the demonstrated needs of patients, payers/ purchasers of health research, peer review editors, policy makers, principal investigators, product makers, program managers, providers and the public [2]. With more than 8000 systematic reviews in the Cochrane Database of Systematic Reviews, authored by individuals from over 50 countries, it is widely accepted that the prioritisation of initiating new reviews and updating published reviews with new evidence is vital. This is reflected by the introduction of mandatory priority setting standards in Cochrane in 2019 [3].

In 2020, Cochrane leadership acknowledged that whilst making priority setting mandatory for all CRGs was an important first step, this approach had not necessarily created a global focus, nor encouraged collaborative priority setting processes across CRGs. As a result, the CRG Networks Priority Setting Working Group was formed. This Group aimed to complement the priority setting work of CRGs by instigating a broad perspective to priority setting across the entire health sector. Reducing global health inequities, defined as avoidable and unfair differences in health [4], was selected as the initial focus for the Group, given the increasing focus on health equity as reflected in the Commission on Social Determinants of Health [5], the United Nations (UN) Millennium Development Goals [6] and the UN Sustainable Development Goals [7].

Systematic reviews have long been criticised for failing to address effects on health equity [8, 9], hindering their applicability to priority populations. Few Cochrane reviews have concentrated on health equity-focused issues or included health equity aspects, and of those that have, many use varying methodology and often lack transparent reporting [10]. This is understandable, as the methods for addressing health equity in systematic reviews have only recently been formalised. In 2020, a chapter concerning health equity was included for the first time in the Cochrane Handbook version 6.1 [4] and a series of health equity training modules were added to the Cochrane online interactive learning platform [11]. Additionally, the Methodological Expectations of Cochrane Intervention Reviews (conduct standard 4) [12] now specifies that it is highly desirable for review authors to ‘consider in advance whether issues of equity are important to the review, and plan for appropriate methods to address them such as those relating to particular participant groups (low-socioeconomic groups, low- or middle-income regions, women, children and older people), intervention comparisons or outcome’.

The consideration of health equity in systematic reviews can be achieved through employment of a ‘health equity lens’, defined by the Campbell and Cochrane Equity Methods Group as ‘a focus on health equity to ensure that the most hard-to-reach groups within a population benefit, while avoiding intervention-generated inequalities’ [4]. The term ‘health equity lens’ is used herein. Approaches for applying a health equity lens include assessing the effects of interventions in disadvantaged populations or in the general population whilst considering characteristics for potential disadvantage, or assessing the effects of interventions aimed at reducing social gradients [4]. The Campbell and Cochrane Equity Methods Group recommends that review authors should look for differences in baseline risk or intervention effectiveness and implementation by characteristics denoted by the acronym ‘PROGRESS-Plus’[13]. ‘PROGRESS’ refers to: place of residence, race/ ethnicity/ culture/ language, occupation, gender/ sex, religion, education, socio-economic status, and social capital. ‘Plus’ denotes additional factors such as age, sexual orientation, and disability. Being transparent about implications for health equity by considering these characteristics, where feasible, will help to prevent unintentional intervention-generated inequities [14]. It will ensure systematic review evidence is relevant to health decision makers worldwide, who are under increasing pressure from their constituents to address health inequities.

This paper reports the methodology and results of a pilot priority setting exercise involving a group of international stakeholders. The aim of this exercise was to prioritise Cochrane reviews of interventions to be updated with a health equity lens, where it is important to understand the distribution of effects across one or more PROGRESS-Plus characteristics.

## Methods

The original intent for this project was to make recommendations on how a health equity lens could be applied to country and regional level improvements or deterioration in both the mortality and morbidity components included in Disability-Adjusted Life Years (DALY). The DALY metric is used in the World Health Organisation (WHO) Global Burden of Disease Project [15] and in the assessment of the success of Universal Health Coverage [16]. However, to pilot the methodology with limited resources, the first phase reported here focused on mortality as the primary outcome.

The Universal Health Coverage Measurement framework was used to identify conditions with a high burden of disease for inclusion in this project [16]. The framework builds on the 2014 WHO and World Bank Framework for Universal Health Coverage and uses WHO Global Burden of Disease 2019 project data [15]. It outlines needed health services across the life course, while accounting for potential health gains delivered to populations. It has mapped 23 conditions across health service types and population age groups for 204 countries and territories from 1990 to 2019. This was deemed a useful starting point for the present project, as the framework identified these conditions through a robust consultation process, and the set of conditions aims to represent a variety of health services that populations need across their lifespans.[16] The methods section of the framework states “For effectiveness, incremental values were assumed by category… as informed by studies published in the Cochrane Database of Systematic Reviews” [16], emphasising the importance of these Cochrane reviews being up to date. This project was also informed by the Equity Effectiveness Loop, developed by the Campbell and Cochrane Equity Methods Group [17]. It measures the burden of illness and effectiveness of interventions across social, demographic, and geographic factors in which disadvantage might exist.

In line with the Equity Effectiveness Loop [17], we first considered the burden of illness by focusing on 21 conditions from the Universal Health Coverage measurement framework [16]. We excluded two conditions from the framework due to having limited resources, outlined further below. We added malaria and 20 neglected tropical diseases (NTDs), as specified by WHO [18], giving 42 conditions to focus on, as shown in Table 1.

**Table 1:** 42 health conditions considered in this priority setting exercise

Effective Coverage Indicators from the Universal Health Coverage Measurement Framework
Tuberculosis treatment
Acute lymphoid leukaemia treatment
Breast cancer treatment
Cervical cancer treatment
Uterine cancer treatment
Colon and rectum cancer treatment
Ischaemic heart disease treatment
Stroke treatment
Diabetes treatment
Chronic kidney disease treatment
Chronic obstructive pulmonary disease treatment
Asthma treatment
Epilepsy treatment
Diarrhoea treatment
Lower respiratory infections treatment
Appendicitis treatment
Paralytic ileus and intestinal obstruction treatment
Antiretroviral therapy coverage
Met need for family planning with modern contraception
Measles-containing-vaccine coverage, 1 dose
Diphtheria-tetanus-pertussis vaccine coverage, 3 doses
**Additional Conditions**
Malaria
Buruli ulcer
Chagas disease
Dengue and Chikungunya
Dracunculiasis (guinea-worm disease)
Echinococcosis
Foodborne trematodiases
Human African trypanosomiasis (sleeping sickness)
Leishmaniasis
Leprosy (Hansen’s disease)
Lymphatic filariasis
Mycetoma, chromoblastomycosis and other deep mycoses
Onchocerciasis (river blindness)
Rabies
Scabies and other ectoparasites
Schistosomiasis
Soil-transmitted helminthiases
Snakebite envenoming
Taeniasis/Cysticercosis
Trachoma
Yaws (Endemic treponematoses)

The protocol for this project was shared with all members of the CRG Networks in March 2021 and can be found on the Cochrane Priority Setting web page [19]. Due to resource limitations, there were three differences between the protocol and the final project:

1. We focused on 42 conditions rather than 44 as stated in the protocol. We excluded two effective coverage indicators from the Universal Health Coverage measurement framework: ‘antenatal, peri-partum, and postnatal care for new-born babies’ and ‘antenatal, postpartum, and postnatal care for mothers’. We decided inclusion of these major topics would result in an unmanageable number of reviews for the pilot and should be addressed in the next phase of equity priority setting.
2. After prioritisation, we did not discuss feasibility of review completion within the Group to arrive at a final list of 10 reviews to be updated, instead we shared the prioritised list of 33 reviews with CRG Network Senior Editors and liaised with Cochrane management regarding the results and future plans.
3. We did not evaluate the project.

Our methodology is outlined in steps 1–4.

### Step 1: map Cochrane reviews assessing mortality to the chosen health conditions responsible for major inequities globally

In November 2020, one researcher (ET) searched Cochrane’s Editorial Management System for active Cochrane systematic reviews concerning the 42 conditions in Table 1 that had assessed mortality and included at least one Summary of Findings table. We only included reviews featuring a Summary of Findings table to assist us in identifying reviews that include an effect on mortality and to extract effect sizes. It also allowed us to focus on reviews published more recently (Summary of Findings tables were introduced in mid 2010s). Any queries were discussed with another researcher (JPP). The search strategy is shown in Additional File Sect. 1. We downloaded information about the reviews into an Excel spreadsheet. Details concerning the number of reviews identified are located in Additional File Sect. 2.

### Step 2: reduce the list of Cochrane reviews by exploring the effectiveness of interventions

We extracted effect sizes (including risk ratios, hazard ratios and odds ratios) for mortality from reviews if it was within the effect size threshold < 0.67 or > 1.5. The team chose this threshold as showing a meaningful effect over a large population [20]. If reviews had more than one mortality effect size, we selected the greatest effect size to represent the review. We then assessed abstracts and retained only reviews featuring a benefit on mortality. If this was not clear from the abstract, we looked at events and study samples stated in the Summary of Findings table.

Reviews of interventions that were found to be effective were prioritised in this pilot project. A well known phenomenon is the funnel of attrition, when different contextual factors might reduce the effect of the intervention in different populations, especially vulnerable populations.[21]. We felt end-users will be more interested in demonstrating whether the benefit of effective interventions remains in disadvantaged populations, and it was thought that many systematic reviews that showed no benefit across all populations would show previously unrecognised benefit in disadvantaged populations.

### Step 3: work with key stakeholders and partners to prioritise reviews for update

We invited 14 stakeholders, defined as ‘an individual or group who is responsible for or affected by health- and healthcare-related decisions that can be informed by research evidence’ [2], to take part in the priority setting exercise. We used purposive selection (use of known contacts) whilst aiming to create a diverse and balanced sample [22] of people from Cochrane Groups and partner organisations. We used this approach to maximise the likelihood of keeping people engaged, to ensure feasibility of the pilot, and because we required input from Cochrane groups who were already known to us.

Our sample of 14 stakeholders included representation from 8 Cochrane Review Group Networks, 1 Cochrane Field (Cochrane Consumer Network), 1 Cochrane Geographic Group (Cochrane Argentina), 3 relevant organisations (Pan American Health Organisation (PAHO), Evidence Aid and the Campbell Collaboration) and 3 health equity experts. Everyone agreed to take part and we held teleconferences using Zoom to outline the project. The sample was 50% female and individuals were located in Argentina, Cameroon, Canada, Germany, India, Lebanon, South Africa, United Kingdom, and the United States of America.

We divided stakeholders into two groups (Team A and Team B) ensuring a balance of location, sex and groups represented. We then randomised the sample of 33 reviews between the groups using rows in an Excel spreadsheet, giving ‘Team A’ 16 reviews and ‘Team B’ 17 reviews. This was decided in conjunction with stakeholders via teleconference, to make the task more manageable. We asked people to independently rank their assigned list of reviews with 1 equalling highest priority for update and 16 or 17 (depending on which team) as the lowest priority.

We selected a subset of 12 items that focus on inequities from the SPARK tool for priority setting [23] for stakeholders to consider during prioritisation (Additional File Sect. 3). The SPARK tool was developed for the prioritisation of reviews in health policy and systems research and includes 13 items to be rated by policy makers and stakeholders and another 9 items to be rated by review teams. We included equity-focused items such as ‘addressing this question responds to global health priorities’ and ‘addressing this question is expected to positively impact health equity’. We asked people to consider these items when prioritising reviews, but we did not require completion of the tool.

On 1st April 2021, we sent stakeholders an Excel spreadsheet containing the list of reviews for ranking (including a link to the review and date of publication), the modified SPARK tool for information, and presentation slides from the introductory teleconference and the project protocol. We asked stakeholders to rank the reviews independently and return rankings within 4 weeks.

### Step 4: consolidation of rankings

For Team A, 16 reviews were scored by 7 stakeholders generating a score for each review between 7 and 112 and the corresponding percentage of the total score was calculated for each review (i.e., score/112 × 100), yielding the overall priority score. Similarly, for Team B, 17 reviews were scored by 6 stakeholders (one stakeholder withdrew from the project due to conflicting demands) generating a score for each review between 6 and 102 and the percentage of the total score (i.e., score/102 × 100) for each review was calculated, yielding the overall priority score. The overall priority scores for Team A and B were amalgamated into a single listing ordered by the score (lowest score = highest priority). The results of the priority setting exercise were fed back to Senior Editors for the CRG Networks and Cochrane management.

### Patient and public involvement

This project involved one stakeholder (EEAN) who identifies as a representative of the patient and public stakeholder group, and who was taking part as a representative of the Cochrane Consumer Network (a community of patients, carers, family members and others who are interested in reliable health evidence). The limited involvement of patient and public members in this project was due to a lack of resources to conduct the pilot exercise and due to the variety of conditions being considered. The stakeholder group involved in the project was balanced across Cochrane Groups, health equity experts and relevant external organisations. It was felt this was appropriate for the pilot, to be able to test the methodology in Cochrane and complete the project within the timelines and with available resources. However, the research team recognises that patient and public involvement is important in priority setting, and aims to involve more members of this stakeholder group in future exercises.

The international stakeholders and researchers who took part in this project, including the patient and public representative, commented on the design of the study following the introductory teleconference and in line with this, methods were amended (e.g. splitting the number of reviews stakeholders would assess, to reduce the burden and time on stakeholders) and finalised. All stakeholders were involved in prioritising the reviews and were invited to provide input to this manuscript as co-authors, to which eleven accepted, providing valuable input.

## Results

### Step 1: map Cochrane reviews assessing mortality to the chosen health conditions responsible for major inequities globally

We identified 359 reviews concerning the conditions in Table 1 that assessed mortality and included at least one Summary of Findings table. We found no reviews meeting our criteria for 13/42 conditions (mostly neglected tropical diseases): Dracunculiasis, Foodborne trematodiases, Measles-containing-vaccine coverage, Buruli ulcer, Echinococcosis, Human African trypanosomiasis (sleeping sickness), Leishmaniasis, Leprosy (Hansen’s disease), Mycetoma/ chromoblastomycosis and other deep mycoses, Rabies, Scabies/ other ectoparasites, Snakebite envenoming, Taeniasis/ Cysticercosis. Additional File Sect. 2 shows the results of this search.

### Step 2: reduce the list of Cochrane reviews by exploring the effectiveness of interventions

After reducing the list of reviews to those showing effective and beneficial interventions, we had 105 reviews. As shown in Table 2, these were spread across 19/42 conditions being considered in this pilot. The reviews were from 16/52 CRGs and a review group featured from each of Cochrane’s 8 CRG Networks.

**Table 2:** 105 Cochrane reviews with meaningful mortality effect size focusing on the prespecified conditions

19 conditions	Number of Cochrane Reviews with mortality reduction and effect size < 0.67 or > 1.5
Acute lymphoid leukaemia treatment	1
Antiretroviral therapy coverage	3
Asthma	4
Breast Cancer	2
Cervical Cancer	3
Chagas disease	1
Chronic kidney disease	16
Chronic obstructive pulmonary disease treatment	12
Colon and rectum cancer treatment	5
Diabetes	10
Diarrhoea treatment	1
Diphtheria-tetanus-pertussis vaccine coverage	1
Epilepsy	1
Ischaemic heart disease	7
Lower respiratory infections	7
Malaria	8
Schistosomiasis	1
Stroke	16
Tuberculosis	6

After exploring whether reviews reported a beneficial effect regarding mortality, we had a sample of 33 reviews to prioritise. The review titles are shown in Table 3, alongside the condition they map to and the year each review was published. The references for these reviews are given in Additional File Sect. 4.

**Table 3:** Final set of 33 Cochrane reviews for prioritisation

13 conditions for which we found reviews	No. reviews	Review title*	Year of current review version
Antiretroviral therapy coverage	2	Antiretroviral therapy (ART) for treating HIV infection in ART-eligible pregnant women	2010
		Optimal time for initiation of antiretroviral therapy in asymptomatic, HIV-infected, treatment-naive adults	2010
Breast Cancer	2	Primary prophylactic colony-stimulating factors for the prevention of chemotherapy-induced febrile neutropenia in breast cancer patients	2012
		Trastuzumab containing regimens for early breast cancer	2012
Cervical Cancer	3	Adjuvant platinum-based chemotherapy for early stage cervical cancer	2016
		Comparison of different human papillomavirus (HPV) vaccine types and dose schedules for prevention of HPV-related disease in females and males	2019
		Extended-field radiotherapy for locally advanced cervical cancer	2018
Chagas Disease	1	Trypanocidal drugs for chronic asymptomatic Trypanosoma cruzi infection	2014
Chronic kidney disease	3	Interventions for idiopathic steroid-resistant nephrotic syndrome in children	2019
		Immunosuppressive treatment for primary membranous nephropathy in adults with nephrotic syndrome	2021
		Phosphate binders for preventing and treating chronic kidney disease-mineral and bone disorder (CKD-MBD)	2018
Chronic obstructive pulmonary disease treatment	5	Hospital at home for acute exacerbations of chronic obstructive pulmonary disease	2012
		Indacaterol, a once-daily beta2-agonist versus twice-daily beta2-agonists or placebo for chronic obstructive pulmonary disease	2015
		Antibiotics for exacerbations of chronic obstructive pulmonary disease	2018
		Non-invasive ventilation for the management of acute hypercapnic respiratory failure due to exacerbation of chronic obstructive pulmonary disease	2017
		Oxygen therapy in the pre-hospital setting for acute exacerbations of chronic obstructive pulmonary disease	2020
Colon and rectum cancer treatment	2	Strategies for detecting colon cancer in patients with inflammatory bowel disease	2017
		Second-line systemic therapy for metastatic colorectal cancer	2017
Diphtheria-tetanus-pertussis vaccine coverage	1	Vaccines for women for preventing neonatal tetanus	2015
Ischaemic heart disease	2	Exercise-based cardiac rehabilitation for coronary heart disease	2021
		Hyperbaric oxygen therapy for acute coronary syndrome	2015
Lower respiratory infections	3	Adjunctive corticosteroids for Pneumocystis jiroveci pneumonia in patients with HIV infection	2015
		Corticosteroids for pneumonia	2017
		Prophylaxis for Pneumocystis pneumonia (PCP) in non-HIV immunocompromised patients	2014
Malaria	5	Intermittent preventive treatment for malaria in children living in areas with seasonal transmission	2012
		Artemether for severe malaria	2019
		Artesunate versus quinine for treating severe malaria	2012
		Home- or community‐based programmes for treating malaria	2013
		Insecticide-treated nets for preventing malaria	2018
Stroke	2	Carotid endarterectomy for symptomatic carotid stenosis	2020
		Peroxisome proliferator-activated receptor gamma agonists for preventing recurrent stroke and other vascular events in people with stroke or transient ischaemic attack	2019
Tuberculosis	2	Interventions for treating tuberculous pericarditis	2017
		Isoniazid for preventing tuberculosis in HIV-infected children	2017

### Step 3: work with key stakeholders and partners to prioritise reviews for update

One person from ‘Team B’ withdrew from the project due to conflicting demands. All other stakeholders completed the prioritisation exercise. This resulted in there being 7 people in Team A (assigned 16 reviews) and 6 people in Team B (assigned 17 reviews).

### Step 4: consolidation of rankings

The results of the priority setting exercise are shown in Table 4. Reviews are ordered in the table from highest overall priority (lowest score) to lowest overall priority (highest score) for update with a health equity focus.

**Table 4:** Results of prioritising reviews for update with a health equity focus

Review Title	Condition	Overall priority score
Intermittent preventive treatment for malaria in children living in areas with seasonal transmission	Malaria	11
Antiretroviral therapy (ART) for treating HIV infection in ART-eligible pregnant women	Antiretroviral therapy coverage	16
Isoniazid for preventing tuberculosis in HIV-infected children	Tuberculosis	20
Artesunate versus quinine for treating severe malaria	Malaria	23
Corticosteroids for pneumonia	Lower respiratory infections	24
Home- or community‐based programmes for treating malaria	Malaria	26
Vaccines for women for preventing neonatal tetanus	Diphtheria-tetanus-pertussis vaccine coverage	33
Insecticide-treated nets for preventing malaria	Malaria	38
Exercise-based cardiac rehabilitation for coronary heart disease	Ischaemic heart disease	44
Interventions for idiopathic steroid-resistant nephrotic syndrome in children	Chronic kidney disease	48
Hospital at home for acute exacerbations of chronic obstructive pulmonary disease	Chronic obstructive pulmonary disease treatment	48
Optimal time for initiation of antiretroviral therapy in asymptomatic, HIV-infected, treatment-naive adults	Antiretroviral therapy coverage	48
Antibiotics for exacerbations of chronic obstructive pulmonary disease	Chronic obstructive pulmonary disease treatment	49
Trypanocidal drugs for chronic asymptomatic Trypanosoma cruzi infection	Chagas Disease	51
Trastuzumab containing regimens for early breast cancer	Breast Cancer	53
Artemether for severe malaria	Malaria	57
Interventions for treating tuberculous pericarditis	Tuberculosis	58
Second-line systemic therapy for metastatic colorectal cancer	Colon and rectum cancer treatment	59
Carotid endarterectomy for symptomatic carotid stenosis	Stroke	60
Non-invasive ventilation for the management of acute hypercapnic respiratory failure due to exacerbation of chronic obstructive pulmonary disease	Chronic obstructive pulmonary disease treatment	61
Adjunctive corticosteroids for Pneumocystis jiroveci pneumonia in patients with HIV infection	Lower respiratory infections	62
Prophylaxis for Pneumocystis pneumonia (PCP) in non-HIV immunocompromised patients	Lower respiratory infections	62
Extended-field radiotherapy for locally advanced cervical cancer	Cervical Cancer	67
Immunosuppressive treatment for primary membranous nephropathy in adults with nephrotic syndrome	Chronic kidney disease	67
Comparison of different human papillomavirus (HPV) vaccine types and dose schedules for prevention of HPV-related disease in females and males	Cervical Cancer	70
Indacaterol, a once-daily beta2-agonist, versus twice-daily beta2-agonists or placebo for chronic obstructive pulmonary disease	Chronic obstructive pulmonary disease treatment	70
Strategies for detecting colon cancer in patients with inflammatory bowel disease	Colon and rectum cancer treatment	70
Hyperbaric oxygen therapy for acute coronary syndrome	Ischaemic heart disease	72
Primary prophylactic colony-stimulating factors for the prevention of chemotherapy-induced febrile neutropenia in breast cancer patients	Breast Cancer	73
Adjuvant platinum-based chemotherapy for early stage cervical cancer	Cervical Cancer	74
Peroxisome proliferator-activated receptor gamma agonists for preventing recurrent stroke and other vascular events in people with stroke or transient ischaemic attack	Stroke	75
Oxygen therapy in the pre-hospital setting for acute exacerbations of chronic obstructive pulmonary disease	Chronic obstructive pulmonary disease treatment	78
Phosphate binders for preventing and treating chronic kidney disease-mineral and bone disorder (CKD-MBD)	Chronic kidney disease	79

Lowest score = highest overall priority

## Discussion

### Summary of main results

This project has developed a methodology to set priorities for updating systematic reviews with a health equity lens, across a wide range of health conditions. Adhering to the mandatory standards for priority setting specified by Cochrane [3] and involving international stakeholders, the pilot identified and prioritised 33 reviews pertaining to health conditions with a high global burden of disease and for which interventions have a meaningful, beneficial impact on mortality. The broad topic areas of the 33 reviews included antiretroviral therapy coverage, cancers, neglected tropical diseases, chronic kidney disease, chronic obstructive pulmonary disease, stroke, ischaemic heart disease, tuberculosis, malaria, lower respiratory infections, and diphtheria-tetanus-pertussis vaccination.

The development of a methodology to prioritise equity in systematic reviews is timely given the pressing need to ensure research evidence is available and relevant to everyone, and the increasing focus on health equity in research. By identifying reviews of high priority to be updated with a health equity lens in this project, the Campbell and Cochrane Equity Methods Group can focus its efforts to provide methodological support to those responsible for updating these reviews. Many of the prioritised reviews have not been updated in the past five years and some within the last ten years, emphasising the importance of considering health equity in the prioritisation of review updates.

The methodology developed in this project may also be useful to organisations outside of Cochrane and the Campbell and Cochrane Equity Methods Group is interested in collaborating with others to prioritise consideration of equity in evidence synthesis. The Global Commission on Evidence to Address Societal Changes draws attention to the importance of considering equity when generating evidence across a range of living evidence formats to address societal challenges, and includes a chapter on equity considerations in its recent report [24]. The Global Evidence Synthesis Initiative (GESI) works to improve the capacity for research synthesis through its Network of 47 centres in 25 low and middle-income countries (LMICs) which provides a platform to exchange evidence synthesis skills and opportunities, and to collaborate on multi-institutional and cross disciplinary evidence synthesis projects [25, 26]. The International Development Coordinating Group (IDCG) in the Campbell Collaboration produces systematic reviews of policy focusing on interventions in LMICs [27]. The Joanna Briggs Institute’s Manual for Evidence Synthesis encourages the use of mixed methods systematic reviews that incorporate the different types of information guideline developers need when making a decision, including impact on equity [28].

There are also exciting developments in applying an equity lens to research mapping. The International Initiative for Impact Evaluation uses mapping approaches to highlight existing evidence on equity, with the ‘3ie Development Evidence Portal’ allowing users to freely search for impact evaluations and systematic reviews and filter by equity considerations [29]. Epistemonikos, one of the largest databases of health evidence, is also prioritising equity as part of the freely accessible classification platform of the Living OVerview of Evidence (L-OVE) project, via coding articles by PROGRESS-Plus factors, with the aim of opening the platform up for use by any organisation at the end of 2023. This will provide an easy way for decision-makers and systematic reviewers to consider equity by quickly being able to retrieve relevant articles, filtered by equity factors [30]. Likewise, the Campbell Collaboration is undertaking mapping initiatives designed to include interventions that target vulnerable groups or aim to reduce inequalities, or impact disadvantaged groups by analysing outcomes specific to vulnerable groups [31, 32].

Furthermore, the COVID-19 pandemic has brought health equity to the forefront of research priorities by worsening the vast social injustices in health. The UN Research Roadmap for COVID-19 recovery has prioritised 25 research areas and scientific strategies to support a recovery from COVID-19 that promotes equity and benefits everyone [33]. The eCOVID-19RecMap is a living list of guidelines which contains the best available recommendations on COVID-19 and highlights equity by allowing users to filter responses by PROGRESS-Plus factors [34]. The COVID Evidence Network to support Decision making (COVID-END) [35], which aims to facilitate access to the best available evidence and reduce evidence duplication regarding COVID-19, has an Equity Task Group which highlights ways equity can be considered. For example, the Group recently produced guidance for considering equity in the context of rapid evidence synthesis [36]. Furthermore, Evidence Aid has introduced a section to its plain language summaries of systematic reviews to outline any consideration of equity in the summarised review, arising from the development of a new evidence collection on resilient health systems, in partnership with PAHO. It will be critical for evidence synthesis organisations to prioritise equity in reviews relevant to COVID-19, and the methodology developed in this paper could be adapted for this purpose.

### Strengths, limitations and ideas for future research

This project demonstrates that it is possible to undertake a priority setting exercise involving an international group of stakeholders online and with limited resources. Following initial introductory teleconferences, the stakeholders ranked reviews independently and submitted responses by email. Despite our group of stakeholders being diverse, we acknowledge that these individuals are not representative of all stakeholders and a more expansive group would have been optimal.

Our use of the Universal Health Coverage framework, recently updated with Global Burden of Disease estimates, facilitated identification of conditions contributing to health inequities for which interventions are effective. We expanded on this framework to include malaria and neglected tropical diseases. Limitations related to using the Universal Health Coverage framework include that it only focused on currently measurable conditions for which sufficient data across countries and methods for measurement exist[16]. Searching using Cochrane’s Editorial Management System allowed us to efficiently identify reviews that included mortality and download spreadsheets of reviews pertaining to specific conditions. It is possible that by including only reviews that featured a Summary of Findings table we could have excluded some older reviews that had a large effect and could be of high priority for update with a health equity focus, as Summary of Findings tables were not mandatory for all reviews until the mid-2010s. However, Summary of Findings tables are now accepted as best practice within Cochrane and this choice allowed us to extract mortality effect sizes easily.

Due to resource constraints and this project being a pilot, we focused only on existing reviews of mortality, and we excluded maternal and neonatal reviews from the sample. In addition to the need to extend this pilot to the large number of reviews from the Cochrane Neonatal and Pregnancy and Childbirth Groups that show a meaningful mortality benefit, this pilot should also be extended to apply the equity lens to systematic reviews reporting morbidity outcomes (i.e., for those interventions that have been shown to provide meaningful benefit on morbidity or quality of life), to ensure that universal implementation of these interventions improves equity. This is particularly important, as this project identified no reviews meeting inclusion criteria for 13 conditions – many of which are Neglected Tropical Diseases – a diverse group of 20 conditions affecting more than 1 billion people who live mostly in impoverished communities [18]. This could be linked to the overall lack of funding for research in these health conditions that do not predominantly affect high-income countries, however it is likely that it is also related to the fact that many of these neglected tropical diseases are more associated with morbidity rather than mortality. This further reinforces the importance of extending this project to explore morbidity outcomes, to be able to capture systematic reviews on neglected tropical diseases, which tend to affect the most marginalised populations and are a focus of the Sustainable Development Goals [37].

Lastly, we did not account for the certainty of evidence in this pilot project. However, this may help to streamline the process of updating reviews. For example, there may be instances where an intervention is well established in terms of effectiveness and the effect estimate on mortality has a high level of certainty. In this case, it may be efficient to apply a health equity lens to the review in its current state without updating the search. The certainty of evidence should therefore be considered when implementing this priority setting approach in future.

### Conclusions

The consideration of health equity in systematic reviews is crucial to ensuring that high quality synthesised health evidence is available and applicable to all who need it. The methodology developed in this pilot can be extended to reducing morbidity, and the combination of mortality and morbidity as represented in Disability-Adjusted Life Years and Quality-Adjusted Life Years. The Campbell and Cochrane Equity Methods Group is keen to work with willing authors to update high priority Cochrane reviews which demonstrated meaningful benefit on both mortality and morbidity (or avoidance of harm) with a health equity lens, and to collaborate with other organisations to ensure high-quality evidence is applicable and accessible to health decision makers globally.

## Electronic supplementary material

Below is the link to the electronic supplementary material.

**Additional File**: Additional file containing the steps in the search process, results of step 1 (searching), modified SPARK tool for priority setting, and references for the 33 prioritised reviews.

## Data Availability

The datasets used and/or analysed during the current study are available from the corresponding author on reasonable request.
