# Supplementary material for: Prioritising Cochrane reviews to be updated with health equity focus
Source: Int J Equity Health. 2023 May 5;22:81. doi: 10.1186/s12939-023-01864-z (PMC10161173; doi:10.1186/s12939-023-01864-z)
Supplement: Supplementary file 1 — Additional File: Additional file containing the steps in the search process, results of step 1 (searching), modified SPARK tool for priority setting, and references for the 33 prioritised reviews. [file 12939_2023_1864_MOESM1_ESM.docx]

# Additional File

## 1: Search strategy for Archie to find active reviews with at least one Summary of Findings table assessing mortality

1. Searched for condition via the ‘Editorial and Methods Department’.


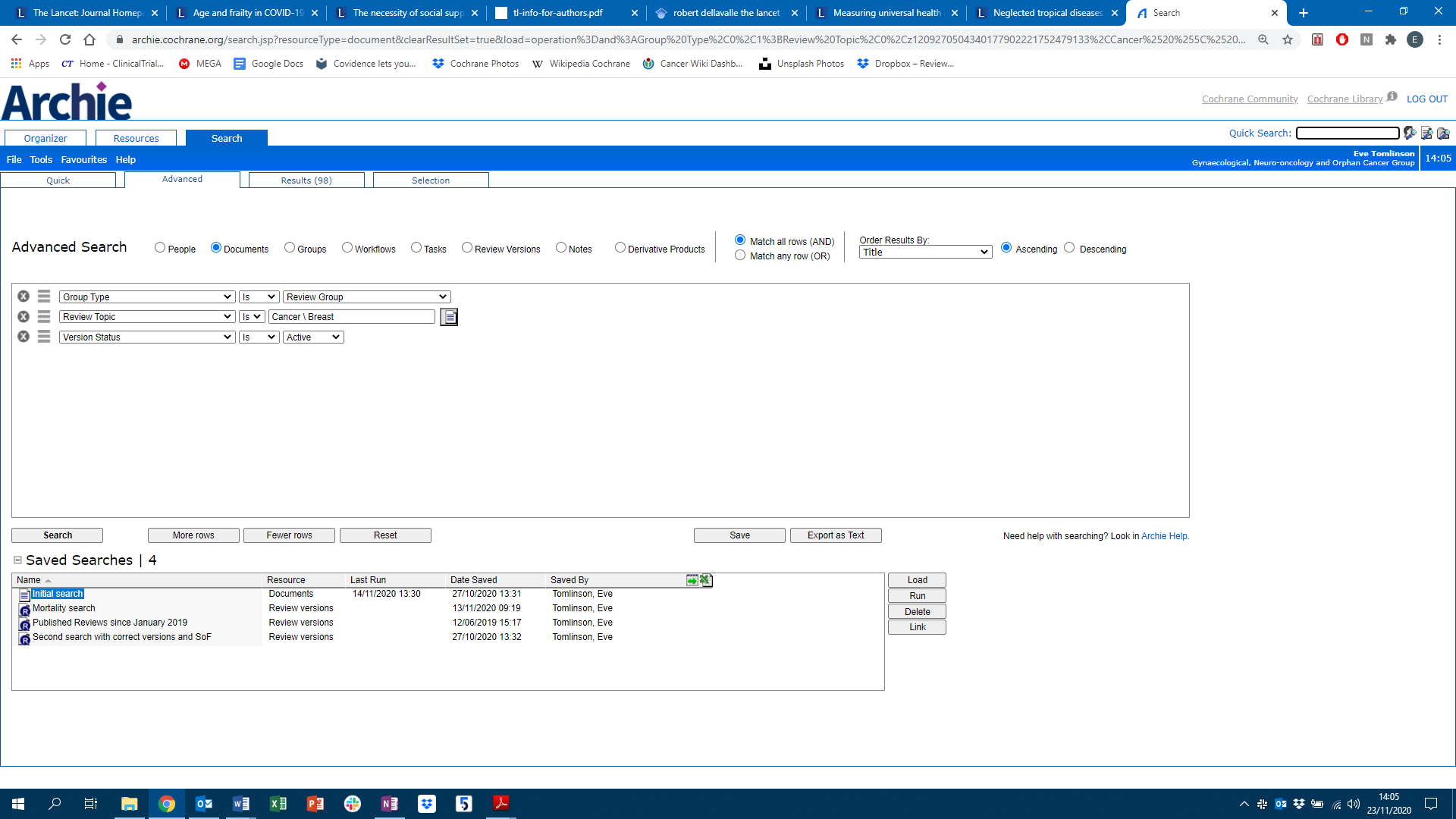


1. Selected all results. This is all active reviews focusing on the selected condition.


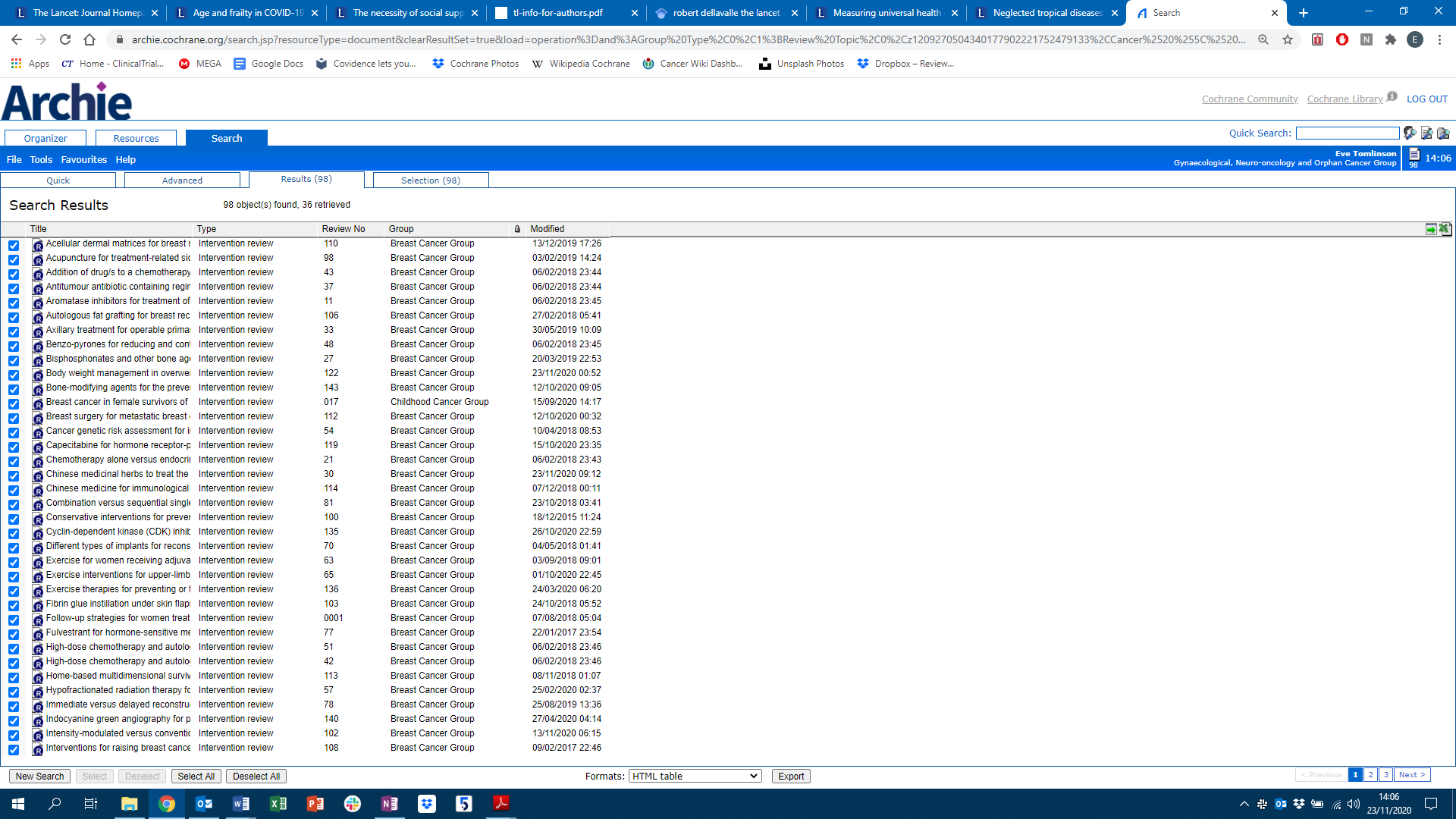


1. Completed a new search to search among the selected reviews to find only reviews with Summary of Findings tables that assess mortality. For all conditions except antenatal/ prenatal we have used the following terms: Mortality, mortality, Death, death, Survival, survival.


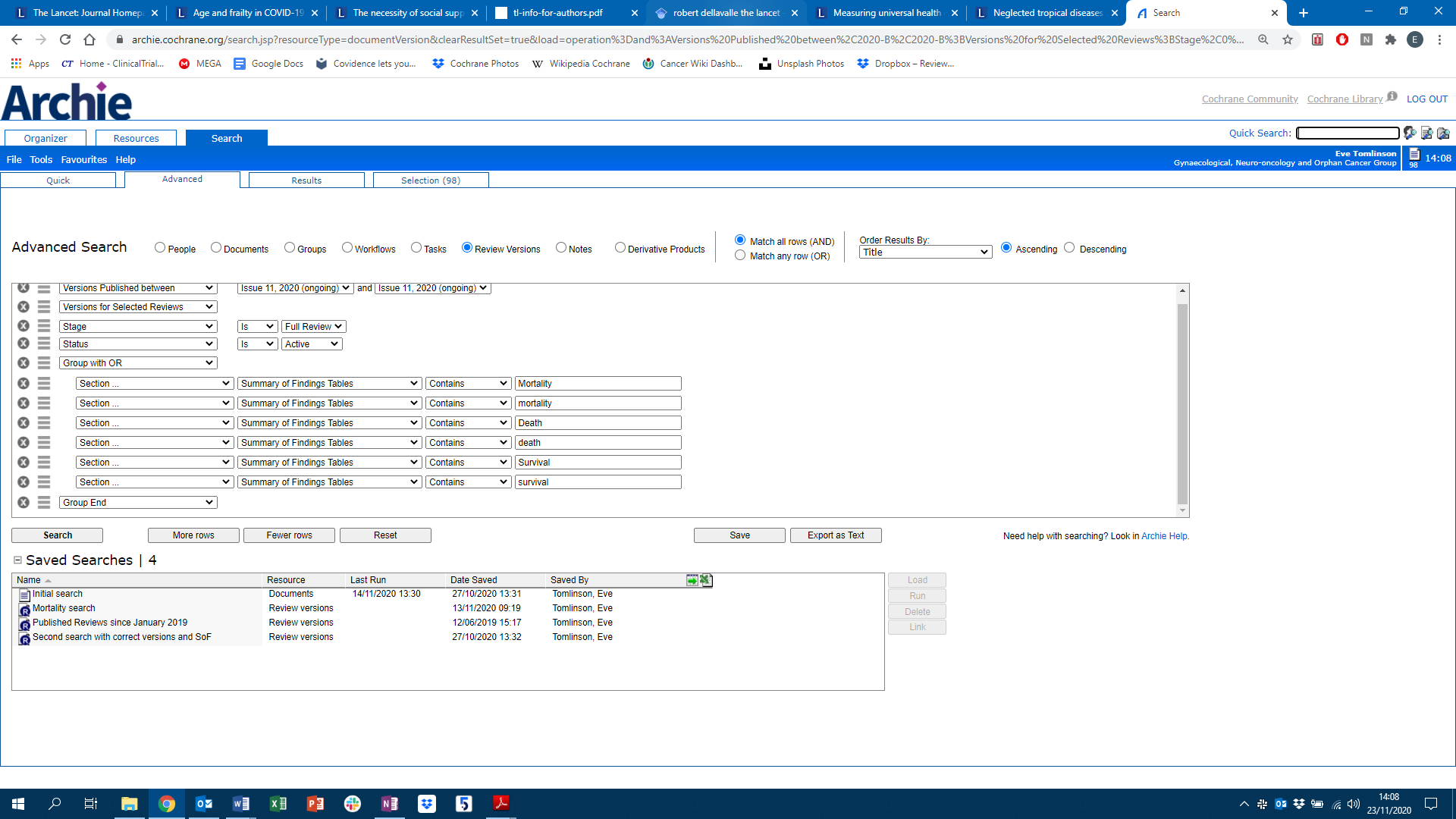


1. Selected all results and export as Excel file including review title, review group, DOI, authors, date searched, update status, abstract, included studies, studies awaiting classification.


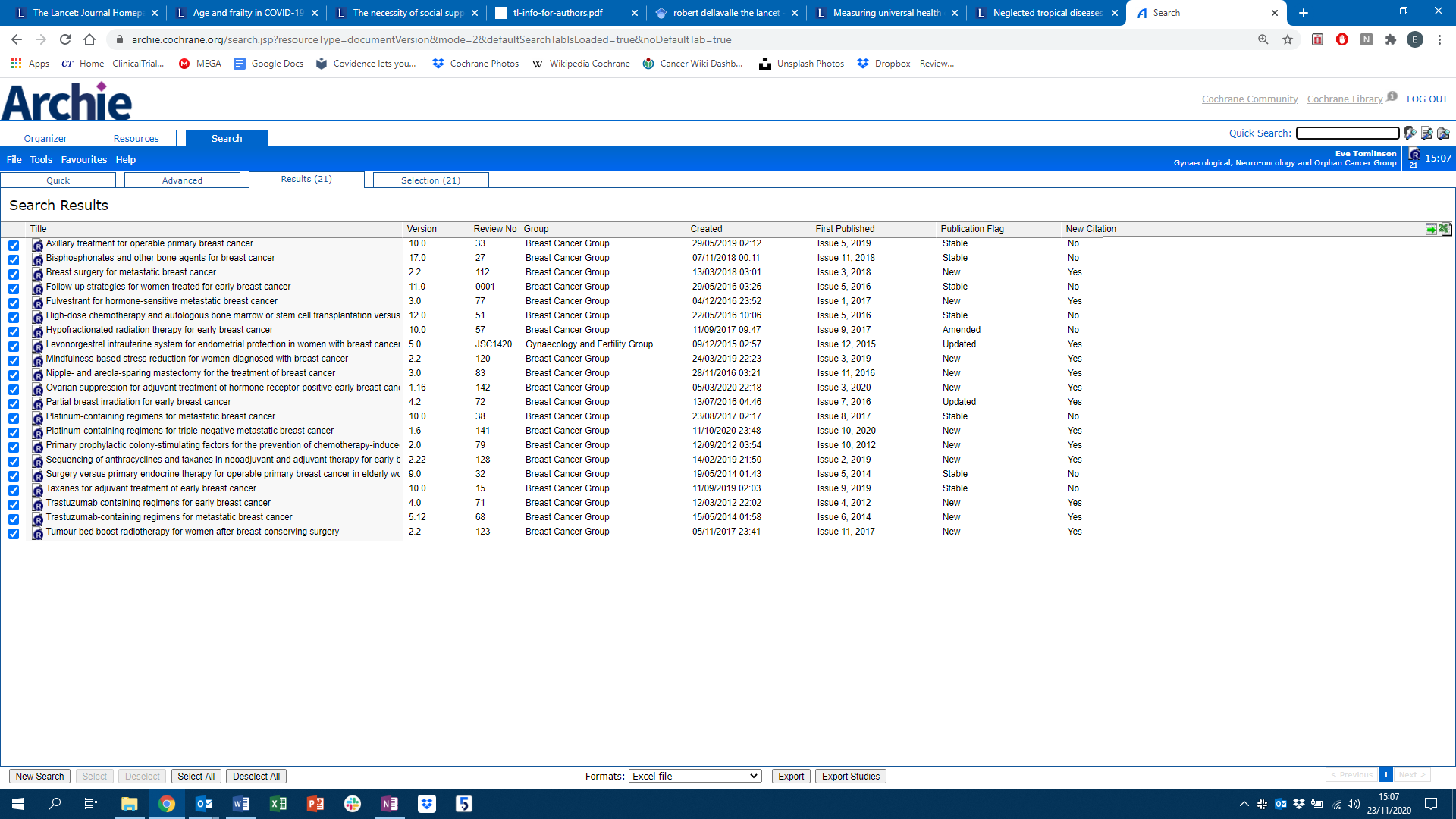


## 2: Results of step 1 – searching Archie to find Cochrane reviews focusing on the chosen health conditions

| **44 Conditions** | **Topic chosen on Archie** | **Reviews from 14/11/2020 search for all active reviews with at least one Summary of Findings table featuring mortality** |
| --- | --- | --- |
| **23 Effective Coverage Indicators from UHC framework** | | |
| Tuberculosis treatment | Infectious diseases 🡪 tuberculosis | 11 reviews |
| Acute lymphoid leukaemia treatment | Blood disorders 🡪 Haematological malignancies 🡪 Acute lymphoblastic leukaemia | 1 review |
| Breast cancer treatment | Cancer 🡪 Breast Cancer | 21 reviews |
| Cervical cancer treatment | Cancer 🡪 Gynaecological 🡪 Cervix | 9 reviews |
| Uterine cancer treatment | Cancer 🡪 Gynaecological 🡪 Uterine body | 9 reviews |
| Colon and rectum cancer treatment | Cancer 🡪 Colorectal | 21 reviews |
| Ischaemic heart disease treatment | Heart & circulation 🡪 Myocardial ischemia/ coronary disease | 28 reviews |
| Stroke treatment | Heart & circulation 🡪 Stroke | 47 reviews |
| Diabetes treatment | Endocrine & metabolic 🡪 Diabetes | 55 reviews |
| Chronic kidney disease treatment | Kidney disease 🡪 Chronic kidney disease | 31 reviews |
| Chronic obstructive pulmonary disease treatment | Spreadsheet has 2 sheets within it:  Lungs & Airways 🡪 Chronic obstructive pulmonary disease (exacerbations), also Chronic obstructive pulmonary disease (stable) | 37 reviews:  8 reviews for exacerbations  29 for stable |
| Asthma treatment | Spreadsheet has 3 sheets within it:  Lungs & Airways 🡪 Asthma (acute), also Asthma (chronic)  Allergy & intolerance 🡪 Allergic asthma | 22 reviews:  5 reviews for acute  16 reviews for chronic  1 for allergic |
| Epilepsy treatment | Neurology 🡪 epilepsy | 7 reviews |
| Diarrhoea treatment | Infectious disease 🡪 diarrhoeal infections | 7 reviews |
| Lower respiratory infections treatment | Spreadsheet has 2 sheets within it:  Child health 🡪 Infectious diseases 🡪 Respiratory infections: general treatment  Child health 🡪 Infectious disease 🡪 Respiratory infections: pneumonia | 13 reviews:  6 reviews for general treatment  7 reviews for pneumonia |
| Appendicitis treatment | Spreadsheet has 2 sheets within it:  Gastroenterology & hepatology 🡪 Other bowel disorders 🡪 appendiceal diseases  Child health 🡪 Gastroenterology 🡪 Appendicitis | 4 reviews:  3 reviews for appendiceal diseases  1 review for child health appencicitis |
| Paralytic ileus and intestinal obstruction treatment | Gastroenterology & Hepatology 🡪 Other bowel disorders 🡪 Bowel obstruction | 1 review |
| Antiretroviral therapy coverage | Infectious disease 🡪 HIV/AIDS 🡪 Treatment: antiretroviral drugs. | 8 reviews |
| Met need for family planning with modern contraception | Reproductive & sexual health 🡪 contraception | 1 review |
| Measles-containing-vaccine coverage, 1 dose | Child health 🡪 Lungs & airways 🡪 Respiratory infections: vaccines  (then discarded non-measles vaccines) | 0 reviews found |
| Diphtheria-tetanus-pertussis vaccine coverage, 3 doses | Infectious disease 🡪 respiratory infections 🡪 diphtheria  Infectious disease 🡪 respiratory infections 🡪 pertussis  (whooping cough)  Infectious disease 🡪 tetanus | 0 reviews for diphtheria  0 reviews for pertussis  1 review for tetanus |
| **21 additional conditions added by project team – malaria and neglected tropical diseases. NGD taken from WHO list:** [**https://www.who.int/neglected_diseases/diseases/en/**](https://www.who.int/neglected_diseases/diseases/en/) | | |
| Malaria | Infectious disease 🡪 Malaria | 13 reviews |
| [Buruli ulcer](https://www.who.int/buruli/en/) | Infectious disease 🡪 Neglected tropical diseases 🡪 Other (there is 1 review on Buruli ulcer in here) | 0 reviews |
| [Chagas disease](https://www.who.int/chagas/en/) | Infectious disease 🡪 Neglected tropical diseases 🡪 Chagas disease (American trypanosomiasis) | 2 reviews |
| [Dengue and Chikungunya](https://www.who.int/denguecontrol/en/) | Infectious disease 🡪 Neglected tropical diseases 🡪 Dengue | 1 review for dengue  Could not find Chikungunya |
| Draculculiasis (guinea- worm disease) | Not downloaded | Not including – Not on Archie and Jordi 5/11 said maybe not needed as on path to eradiaction. |
| [Echinococcosis](https://www.who.int/echinococcosis/en/) | Infectious disease 🡪 Neglected tropical diseases 🡪 [Echinococcosis](https://www.who.int/echinococcosis/en/) | **0 reviews** |
| Foodborne trematodiases | Not downloaded | Not including – Not on Archie and on 5/11 Jordi says maybe not needed as treatment seems set already https://www.who.int/news-room/fact-sheets/detail/foodborne-trematode-infections |
| [Human African trypanosomiasis (sleeping sickness)](https://www.who.int/trypanosomiasis_african/en/) | Infectious disease 🡪 Neglected tropical diseases 🡪 [Human African trypanosomiasis (sleeping sickness)](https://www.who.int/trypanosomiasis_african/en/) | **0 reviews** |
| Leishmaniasis | Infectious disease 🡪 Neglected tropical diseases 🡪 [Leishmaniasis](https://www.who.int/leishmaniasis/en/) | 0 reviews |
| [Leprosy (Hansen's disease)](https://www.who.int/lep/en/) | Infectious disease 🡪 Neglected tropical diseases 🡪 Leprosy | 0 reviews |
| [Lymphatic filariasis](https://www.who.int/lymphatic_filariasis/en/) | Infectious disease 🡪 Neglected tropical diseases 🡪 [Lymphatic filariasis](https://www.who.int/lymphatic_filariasis/en/) | 1 review |
| [Mycetoma, chromoblastomycosis and other deep mycoses](https://www.who.int/neglected_diseases/diseases/mycetoma-chromoblastomycosis-deep-mycoses/en/) | Infectious disease 🡪 Neglected tropical diseases 🡪 Other | 0 reviews |
| [Onchocerciasis (river blindness)](https://www.who.int/onchocerciasis/en/) | Infectious disease 🡪 Neglected tropical diseases 🡪 [Onchocerciasis (river blindness)](https://www.who.int/onchocerciasis/en/) | 1 review |
| [Rabies](https://www.who.int/rabies/en/) | Infectious disease 🡪 Neglected tropical diseases 🡪 [Rabies](https://www.who.int/rabies/en/) | 0 reviews |
| [Scabies and other ectoparasites](https://www.who.int/neglected_diseases/diseases/scabies-and-other-ectoparasites/en/) | Infectious disease 🡪 Neglected tropical diseases 🡪 Scabies | 0 reviews |
| [Schistosomiasis](https://www.who.int/schistosomiasis/en/) | Infectious disease 🡪 Neglected tropical diseases 🡪 [Schistosomiasis](https://www.who.int/schistosomiasis/en/) | 2 reviews |
| [Soil-transmitted helminthiases](https://www.who.int/intestinal_worms/en/) | Infectious disease 🡪 Neglected tropical diseases 🡪 [Soil-transmitted helminthiases](https://www.who.int/intestinal_worms/en/) | 3 reviews |
| [Snakebite envenoming](https://www.who.int/snakebites/en/) | Infectious disease 🡪 Neglected tropical diseases 🡪 Snakebite | 0 reviews |
| [Taeniasis/Cysticercosis](https://www.who.int/taeniasis/en/) | Infectious disease 🡪 Neglected tropical diseases 🡪 [Taeniasis/Cysticercosis](https://www.who.int/taeniasis/en/) | 0 reviews |
| [Trachoma](https://www.who.int/trachoma/en/) | Infectious disease 🡪 Neglected tropical diseases 🡪 Trachoma | 1 review |
| [Yaws (Endemic treponematoses)](https://www.who.int/yaws/en/) | Infectious disease 🡪 Neglected tropical diseases 🡪 Endemic treponematoses | 1 review |
|  |  | **Total: 359 reviews** |

## 3: Modified SPARK Tool for Priority Setting

**In this priority setting pilot we are considering existing Cochrane reviews for update with a health equity focus. For each question, indicate your degree of agreement with each of the following statements by circling the appropriate box.**

1. Addressing this question responds to a problem that is of ***large*** ***burden.***

| Strongly disagree | Disagree | Neither agree nor disagree | Agree | Strongly agree |
| --- | --- | --- | --- | --- |
| 1 | 2 | 3 | 4 | 5 |
| Signaling questions:   - What is the prevalence / incidence of the problem? - What is the associated morbidity and mortality? - What is the associated cost to the healthcare system and/or society at large? | | | | |

1. Addressing this question responds to a problem that is ***persistent.***

| Strongly disagree | Disagree | Neither agree nor disagree | Agree | Strongly agree |
| --- | --- | --- | --- | --- |
| 1 | 2 | 3 | 4 | 5 |
| Signaling questions:  Does the problem pose a continued or recurrent challenge to the healthcare system? | | | | |

1. Addressing this question responds to the ***needs of the population***.

| Strongly disagree | Disagree | Neither agree nor disagree | Agree | Strongly agree |
| --- | --- | --- | --- | --- |
| 1 | 2 | 3 | 4 | 5 |
| Signaling questions:  Does this question align with public expectations? | | | | |

| Strongly disagree | Disagree | Neither agree nor disagree | Agree | Strongly agree |
| --- | --- | --- | --- | --- |
| 1 | 2 | 3 | 4 | 5 |
| Signaling questions:  Does this question align with decision-makers’ expectations? | | | | |

1. Addressing this question responds to the ***needs of decision-makers.***
2. Addressing this question responds to ***global health priorities.***

| Strongly disagree | Disagree | Neither agree nor disagree | Agree | Strongly agree |
| --- | --- | --- | --- | --- |
| 1 | 2 | 3 | 4 | 5 |
| Signaling questions:   - Does this question align with health policies set at a national (or other relevant) level? - Does this question align with existing strategies and plans at a national (or other relevant) level? | | | | |

| Strongly disagree | Disagree | Neither agree nor disagree | Agree | Strongly agree |
| --- | --- | --- | --- | --- |
| 1 | 2 | 3 | 4 | 5 |
| Signaling questions:   - Is the problem being addressed by the question related to human rights? - What are the consequences (e.g., opportunity costs) to the population/society for not addressing this question? | | | | |

1. Addressing this question is a ***moral obligation***.

| Strongly disagree | Disagree | Neither agree nor disagree | Agree | Strongly agree |
| --- | --- | --- | --- | --- |
| 1 | 2 | 3 | 4 | 5 |
| Signaling questions:   - What is the expected number of potential beneficiaries from addressing this question? - Is addressing this question expected to improve population outcomes (e.g., life expectancy, health status, and survival)? - Is addressing this question expected to increase or improve access to services? | | | | |

1. Addressing this question is expected to positively ***impact health equity****.*
2. Addressing this question is expected to positively ***impact population health.***

| Strongly disagree | Disagree | Neither agree nor disagree | Agree | Strongly agree |
| --- | --- | --- | --- | --- |
| 1 | 2 | 3 | 4 | 5 |
| Signaling questions:   - To what extent does addressing this question contribute to horizontal equity (i.e. provision of equal services for people with equal health needs)? - To what extent does addressing this question contribute to vertical equity (i.e. giving priority to disadvantaged groups)? | | | | |

1. Addressing this question is expected to positively ***impact patient experience of care.***

| Strongly disagree | Disagree | Neither agree nor disagree | Agree | Strongly agree |
| --- | --- | --- | --- | --- |
| 1 | 2 | 3 | 4 | 5 |
| Signaling questions:   - Is addressing this question expected to positively impact patient’s expectations of quality of care or services? - Is addressing this question expected to enhance people’s dignity and autonomy, their preferences, and the confidentiality of information? | | | | |

1. Addressing this question is expected to positively ***impact health care expenditures.***

| Strongly disagree | Disagree | Neither agree nor disagree | Agree | Strongly agree |
| --- | --- | --- | --- | --- |
| 1 | 2 | 3 | 4 | 5 |
| Signaling questions:   - Is addressing this question expected to protect people against catastrophic health expenditure? - Is addressing this question expected to decrease unit costs (i.e., total costs per patient from a health systems perspective), and budget impact on health plan? - Is addressing this question expected to decrease financial impact on government? | | | | |

1. Using the research evidence for this question ***is critical to inform decision-making***.

| Strongly disagree | Disagree | Neither agree nor disagree | Agree | Strongly agree |
| --- | --- | --- | --- | --- |
| 1 | 2 | 3 | 4 | 5 |
| Signaling questions:   - Would the research evidence make a difference to the decision-making process? - Can a decision be made without the research evidence? | | | | |

1. Using the research evidence for this question is expected to be ***supported by political actors***.

| Strongly disagree | Disagree | Neither agree nor disagree | Agree | Strongly agree |
| --- | --- | --- | --- | --- |
| 1 | 2 | 3 | 4 | 5 |
| Signaling questions:   - How committed are policymakers and stakeholders to use the research evidence to inform decision-making? - What are the chances of the research evidence being implemented? | | | | |

**Reference for the original SPARK tool, developed by the team at the Center for Systematic Reviews on Health Policy and Systems Research (SPARK) at the American University of Beirut (AUB):** Akl, E. A., Fadlallah, R., Ghandour, L., Kdouh, O., Langlois, E., Lavis, J. N., ... & El-Jardali, F. (2017). The SPARK Tool to prioritise questions for systematic reviews in health policy and systems research: development and initial validation. Health research policy and systems, 15(1), 1-7

## 4: References for the final set of 33 Cochrane reviews for prioritisation

| **Review title** | **Reference** |
| --- | --- |
| Antiretroviral therapy (ART) for treating HIV infection in ART-eligible pregnant women | Sturt AS, Dokubo EK, Sint TT. Antiretroviral therapy (ART) for treating HIV infection in ART‐eligible pregnant women. Cochrane Database of Systematic Reviews 2010, Issue 3. Art. No.: CD008440. DOI: 10.1002/14651858.CD008440. Accessed 23 August 2022. |
| Optimal time for initiation of antiretroviral therapy in asymptomatic, HIV-infected, treatment-naive adults | Siegfried N, Uthman OA, Rutherford GW. Optimal time for initiation of antiretroviral therapy in asymptomatic, HIV‐infected, treatment‐naive adults. Cochrane Database of Systematic Reviews 2010, Issue 3. Art. No.: CD008272. DOI: 10.1002/14651858.CD008272.pub2. Accessed 23 August 2022. |
| Primary prophylactic colony-stimulating factors for the prevention of chemotherapy-induced febrile neutropenia in breast cancer patients | Renner P, Milazzo S, Liu JP, Zwahlen M, Birkmann J, Horneber M. Primary prophylactic colony‐stimulating factors for the prevention of chemotherapy‐induced febrile neutropenia in breast cancer patients. Cochrane Database of Systematic Reviews 2012, Issue 10. Art. No.: CD007913. DOI: 10.1002/14651858.CD007913.pub2. Accessed 23 August 2022. |
| Trastuzumab containing regimens for early breast cancer | Moja L, Tagliabue L, Balduzzi S, Parmelli E, Pistotti V, Guarneri V, D'Amico R. Trastuzumab containing regimens for early breast cancer. Cochrane Database of Systematic Reviews 2012, Issue 4. Art. No.: CD006243. DOI: 10.1002/14651858.CD006243.pub2. Accessed 23 August 2022. |
| Adjuvant platinum-based chemotherapy for early stage cervical cancer | Falcetta FS, Medeiros LRF, Edelweiss MI, Pohlmann PR, Stein AT, Rosa DD, Platt J. Adjuvant platinum‐based chemotherapy for early stage cervical cancer. Cochrane Database of Systematic Reviews 2016, Issue 11. Art. No.: CD005342. DOI: 10.1002/14651858.CD005342.pub4. Accessed 23 August 2022 |
| Comparison of different human papillomavirus (HPV) vaccine types and dose schedules for prevention of HPV-related disease in females and males | Bergman H, Buckley BS, Villanueva G, Petkovic J, Garritty C, Lutje V, Riveros‐Balta AX, Low N, Henschke N. Comparison of different human papillomavirus (HPV) vaccine types and dose schedules for prevention of HPV‐related disease in females and males. Cochrane Database of Systematic Reviews 2019, Issue 11. Art. No.: CD013479. DOI: 10.1002/14651858.CD013479. Accessed 23 August 2022. |
| Extended-field radiotherapy for locally advanced cervical cancer | Thamronganantasakul K, Supakalin N, Kietpeerakool C, Pattanittum P, Lumbiganon P. Extended‐field radiotherapy for locally advanced cervical cancer. Cochrane Database of Systematic Reviews 2018, Issue 10. Art. No.: CD012301. DOI: 10.1002/14651858.CD012301.pub2. Accessed 23 August 2022. |
| Trypanocidal drugs for chronic asymptomatic Trypanosoma cruzi infection | Villar JC, Perez JG, Cortes OL, Riarte A, Pepper M, Marin‐Neto JA, Guyatt GH. Trypanocidal drugs for chronic asymptomatic *Trypanosoma cruzi* infection. Cochrane Database of Systematic Reviews 2014, Issue 5. Art. No.: CD003463. DOI: 10.1002/14651858.CD003463.pub2. Accessed 23 August 2022. |
| Interventions for idiopathic steroid-resistant nephrotic syndrome in children | Liu ID, Willis NS, Craig JC, Hodson EM. Interventions for idiopathic steroid‐resistant nephrotic syndrome in children. Cochrane Database of Systematic Reviews 2019, Issue 11. Art. No.: CD003594. DOI: 10.1002/14651858.CD003594.pub6. Accessed 23 August 2022. |
| Immunosuppressive treatment for primary membranous nephropathy in adults with nephrotic syndrome | von Groote TC, Williams G, Au EH, Chen Y, Mathew AT, Hodson EM, Tunnicliffe DJ. Immunosuppressive treatment for primary membranous nephropathy in adults with nephrotic syndrome. Cochrane Database of Systematic Reviews 2021, Issue 11. Art. No.: CD004293. DOI: 10.1002/14651858.CD004293.pub4. Accessed 23 August 2022. |
| Phosphate binders for preventing and treating chronic kidney disease-mineral and bone disorder (CKD-MBD) | Ruospo M, Palmer SC, Natale P, Craig JC, Vecchio M, Elder GJ, Strippoli GFM. Phosphate binders for preventing and treating chronic kidney disease‐mineral and bone disorder (CKD‐MBD). Cochrane Database of Systematic Reviews 2018, Issue 8. Art. No.: CD006023. DOI: 10.1002/14651858.CD006023.pub3. Accessed 23 August 2022. |
| Hospital at home for acute exacerbations of chronic obstructive pulmonary disease | Jeppesen E, Brurberg KG, Vist GE, Wedzicha JA, Wright JJ, Greenstone M, Walters JAE. Hospital at home for acute exacerbations of chronic obstructive pulmonary disease. Cochrane Database of Systematic Reviews 2012, Issue 5. Art. No.: CD003573. DOI: 10.1002/14651858.CD003573.pub2. Accessed 23 August 2022. |
| Indacaterol, a once-daily beta2-agonist versus twice-daily beta2-agonists or placebo for chronic obstructive pulmonary disease | Geake JB, Dabscheck EJ, Wood‐Baker R, Cates CJ. Indacaterol, a once‐daily beta_2_‐agonist, versus twice‐daily beta_2_‐agonists or placebo for chronic obstructive pulmonary disease. Cochrane Database of Systematic Reviews 2015, Issue 1. Art. No.: CD010139. DOI: 10.1002/14651858.CD010139.pub2. Accessed 23 August 2022. |
| Antibiotics for exacerbations of chronic obstructive pulmonary disease | Vollenweider DJ, Frei A, Steurer‐Stey CA, Garcia‐Aymerich J, Puhan MA. Antibiotics for exacerbations of chronic obstructive pulmonary disease. Cochrane Database of Systematic Reviews 2018, Issue 10. Art. No.: CD010257. DOI: 10.1002/14651858.CD010257.pub2. Accessed 23 August 2022. |
| Non-invasive ventilation for the management of acute hypercapnic respiratory failure due to exacerbation of chronic obstructive pulmonary disease | Osadnik CR, Tee VS, Carson‐Chahhoud KV, Picot J, Wedzicha JA, Smith BJ. Non‐invasive ventilation for the management of acute hypercapnic respiratory failure due to exacerbation of chronic obstructive pulmonary disease. Cochrane Database of Systematic Reviews 2017, Issue 7. Art. No.: CD004104. DOI: 10.1002/14651858.CD004104.pub4. Accessed 23 August 2022 |
| Oxygen therapy in the pre-hospital setting for acute exacerbations of chronic obstructive pulmonary disease | Kopsaftis Z, Carson‐Chahhoud KV, Austin MA, Wood‐Baker R. Oxygen therapy in the pre‐hospital setting for acute exacerbations of chronic obstructive pulmonary disease. Cochrane Database of Systematic Reviews 2020, Issue 1. Art. No.: CD005534. DOI: 10.1002/14651858.CD005534.pub3. Accessed 23 August 2022 |
| Strategies for detecting colon cancer in patients with inflammatory bowel disease | Bye WA, Nguyen TM, Parker CE, Jairath V, East JE. Strategies for detecting colon cancer in patients with inflammatory bowel disease. Cochrane Database of Systematic Reviews 2017, Issue 9. Art. No.: CD000279. DOI: 10.1002/14651858.CD000279.pub4. Accessed 23 August 2022. |
| Second-line systemic therapy for metastatic colorectal cancer | Mocellin S, Baretta Z, Roqué i Figuls M, Solà I, Martin‐Richard M, Hallum S, Bonfill Cosp X. Second‐line systemic therapy for metastatic colorectal cancer. Cochrane Database of Systematic Reviews 2017, Issue 1. Art. No.: CD006875. DOI: 10.1002/14651858.CD006875.pub3. Accessed 23 August 2022 |
| Vaccines for women for preventing neonatal tetanus | Demicheli V, Barale A, Rivetti A. Vaccines for women for preventing neonatal tetanus. Cochrane Database of Systematic Reviews 2015, Issue 7. Art. No.: CD002959. DOI: 10.1002/14651858.CD002959.pub4. Accessed 23 August 2022. |
| Exercise-based cardiac rehabilitation for coronary heart disease | Dibben G, Faulkner J, Oldridge N, Rees K, Thompson DR, Zwisler A-D, Taylor RS. Exercise‐based cardiac rehabilitation for coronary heart disease. Cochrane Database of Systematic Reviews 2021, Issue 11. Art. No.: CD001800. DOI: 10.1002/14651858.CD001800.pub4. Accessed 23 August 2022. |
| Hyperbaric oxygen therapy for acute coronary syndrome | Bennett MH, Lehm JP, Jepson N. Hyperbaric oxygen therapy for acute coronary syndrome. Cochrane Database of Systematic Reviews 2015, Issue 7. Art. No.: CD004818. DOI: 10.1002/14651858.CD004818.pub4. Accessed 23 August 2022. |
| Adjunctive corticosteroids for Pneumocystis jiroveci pneumonia in patients with HIV infection | Ewald H, Raatz H, Boscacci R, Furrer H, Bucher HC, Briel M. Adjunctive corticosteroids for Pneumocystis jiroveci pneumonia in patients with HIV infection. Cochrane Database of Systematic Reviews 2015, Issue 4. Art. No.: CD006150. DOI: 10.1002/14651858.CD006150.pub2. Accessed 23 August 2022. |
| Corticosteroids for pneumonia | Stern A, Skalsky K, Avni T, Carrara E, Leibovici L, Paul M. Corticosteroids for pneumonia. Cochrane Database of Systematic Reviews 2017, Issue 12. Art. No.: CD007720. DOI: 10.1002/14651858.CD007720.pub3. Accessed 23 August 2022. |
| Prophylaxis for Pneumocystis pneumonia (PCP) in non-HIV immunocompromised patients | Stern A, Green H, Paul M, Vidal L, Leibovici L. Prophylaxis for Pneumocystis pneumonia (PCP) in non‐HIV immunocompromised patients. Cochrane Database of Systematic Reviews 2014, Issue 10. Art. No.: CD005590. DOI: 10.1002/14651858.CD005590.pub3. Accessed 23 August 2022. |
| Intermittent preventive treatment for malaria in children living in areas with seasonal transmission | Meremikwu MM, Donegan S, Sinclair D, Esu E, Oringanje C. Intermittent preventive treatment for malaria in children living in areas with seasonal transmission. Cochrane Database of Systematic Reviews 2012, Issue 2. Art. No.: CD003756. DOI: 10.1002/14651858.CD003756.pub4. Accessed 23 August 2022. |
| Artemether for severe malaria | Esu EB, Effa EE, Opie ON, Meremikwu MM. Artemether for severe malaria. Cochrane Database of Systematic Reviews 2019, Issue 6. Art. No.: CD010678. DOI: 10.1002/14651858.CD010678.pub3. Accessed 23 August 2022. |
| Artesunate versus quinine for treating severe malaria | Sinclair D, Donegan S, Isba R, Lalloo DG. Artesunate versus quinine for treating severe malaria. Cochrane Database of Systematic Reviews 2012, Issue 6. Art. No.: CD005967. DOI: 10.1002/14651858.CD005967.pub4. Accessed 23 August 2022. |
| Home‐ or community‐based programmes for treating malaria | Okwundu CI, Nagpal S, Musekiwa A, Sinclair D. Home‐ or community‐based programmes for treating malaria. Cochrane Database of Systematic Reviews 2013, Issue 5. Art. No.: CD009527. DOI: 10.1002/14651858.CD009527.pub2. Accessed 23 August 2022. |
| Insecticide-treated nets for preventing malaria | Pryce J, Richardson M, Lengeler C. Insecticide‐treated nets for preventing malaria. Cochrane Database of Systematic Reviews 2018, Issue 11. Art. No.: CD000363. DOI: 10.1002/14651858.CD000363.pub3. Accessed 23 August 2022. |
| Carotid endarterectomy for symptomatic carotid stenosis | Rerkasem A, Orrapin S, Howard DPJ, Rerkasem K. Carotid endarterectomy for symptomatic carotid stenosis. Cochrane Database of Systematic Reviews 2020, Issue 9. Art. No.: CD001081. DOI: 10.1002/14651858.CD001081.pub4. Accessed 23 August 2022. |
| Peroxisome proliferator-activated receptor gamma agonists for preventing recurrent stroke and other vascular events in people with stroke or transient ischaemic attack | Liu J, Wang LN. Peroxisome proliferator‐activated receptor gamma agonists for preventing recurrent stroke and other vascular events in people with stroke or transient ischaemic attack. Cochrane Database of Systematic Reviews 2019, Issue 10. Art. No.: CD010693. DOI: 10.1002/14651858.CD010693.pub5. Accessed 23 August 2022. |
| Interventions for treating tuberculous pericarditis | Wiysonge CS, Ntsekhe M, Thabane L, Volmink J, Majombozi D, Gumedze F, Pandie S, Mayosi BM. Interventions for treating tuberculous pericarditis. Cochrane Database of Systematic Reviews 2017, Issue 9. Art. No.: CD000526. DOI: 10.1002/14651858.CD000526.pub2. Accessed 23 August 2022. |
| Isoniazid for preventing tuberculosis in HIV-infected children | Zunza M, Gray DM, Young T, Cotton M, Zar HJ. Isoniazid for preventing tuberculosis in HIV‐infected children. Cochrane Database of Systematic Reviews 2017, Issue 8. Art. No.: CD006418. DOI: 10.1002/14651858.CD006418.pub3. Accessed 23 August 2022. |
